# Enhancing the Health and Well-Being of People with Chronic Diseases: Assessment and Sustainable Development Planning for Therapeutic Landscapes after Urban Expansion

**DOI:** 10.1155/2021/2828141

**Published:** 2021-10-08

**Authors:** Lin Mei, Kun Liu, Bo-Wei Zhu

**Affiliations:** ^1^School of Art and Design, Wuhan University of Technology, Wuhan, Hubei 430070, China; ^2^School of Art and Design, Quanzhou Normal University, Feng ze, Quanzhou, Fujian 362000, China; ^3^Faculty of Humanities and Arts, Macau University of Science and Technology, Macau 999078, China

## Abstract

Under the influence of economic, environmental, and social structural changes, urban space expands and contracts to varying degrees and the everyday urban landscape changes in response. Over the past 20 years, a large number of cities in China have undergone a brief but rapid urban expansion and are moving toward shrinking cities. Most of these cities are now facing social problems such as an aging population and a high prevalence of chronic diseases. Therefore, the “therapeutic” role and impact of everyday landscapes in these cities need to be examined in the context of urban development processes through appropriate assessment methods. Therefore, this study applies the ANP-mV model to examine the therapeutic nature of everyday urban landscapes in different development periods, with the aim of enhancing the health and well-being of people with chronic diseases. Firstly, this study uses the city of Jinzhou in Northeast China as an example to develop a framework for assessing the therapeutic nature of everyday urban landscapes based on the health care needs of people with chronic diseases; secondly, it examines the therapeutic nature of the former Jinzhou Suburban Riverfront Forest Park as it has developed and evolved over the past 16 years; finally, it explores place-making and regeneration strategies for therapeutic landscapes from the perspectives of dynamic impact and sustainable development to enhance chronic illness patients' well-being. At the theoretical level, this study contributes by providing a methodology and research ideas for examining the “therapeutic” nature of everyday urban landscapes and proposing further development plans for renewal, constructing a framework for assessing therapeutic landscapes, and elucidating the relationship between networks of influence and the relative importance of various assessment dimensions/elements. At the practical application level, the contribution of this study is to provide local policymakers with a key decision basis for the future development planning of the East Lake Forest Park. The aim is to explore landscape creation and regeneration strategies for the East Lake Forest Park in the context of Jinzhou's progressive move toward a shrinking city, in order to sustain the well-being of the chronically ill.

## 1. Introduction

Previous research has repeatedly documented the restorative effects of place on health and well-being [1, 2] and has developed a number of framework concepts such as the biophilia hypothesis, the Attention Restoration Theory, and the concept of healing landscapes [3]. Under the concept of therapeutic landscapes, scholars have examined many types of landscapes associated with healing or rehabilitation, including natural landscapes such as villages, mountains, and lakes that have a reputation for healing [4, 5]. There are also everyday landscapes, such as places and residential areas where medical services are provided, and libraries [6, 7]. Then, there are urban landscapes, such as urban public green spaces and streets, and social networks [8, 9]. In humanist and cultural ecology theories, the formation of therapeutic landscapes is dynamic and the landscape can be seen as an evolving process where “therapeutic” is examined in the context of changing environmental, social, and economic conditions [10]. Yan and He [8] argue that it is important to explore the evolution of therapeutic landscapes, i.e., how therapeutic landscapes change over time.

Realistic experience shows that the development of local productivity, changes in social structure, and the introduction of macrogovernance policies can have a vital impact on the evolution of the urban landscape. When the landscape style changes, it will inevitably lead to discussions about endowing the landscape with healing properties or maintaining the healing properties of the place. Examples include exploring and understanding the creation of restorative and therapeutic spatial places for refugees or dislocated farmers in urban distribution and resettlement planning [9, 11, 12], exploring longevity villages as tourist destinations as the health tourism industry grows [8], and exploring design strategies for healing and therapeutic gardens in senior communities or medical buildings as we move toward a healthy aging society [13]. It is worth noting that as urban economies develop, populations grow, and the quality of life improves, cities at all levels in many regions experience varying degrees of expansion, and the landscape of former suburban areas will evolve significantly under the influence of multiple factors. For local urban dwellers, villages, green spaces, forests, and parks in the suburbs are often seen as therapeutic and healing landscapes that combine physical and nonphysical levels [14, 15].

Due to the economic downturn of cities, industrial transformation, and an aging population, the rate of urban expansion into suburban areas has generally slowed down in recent years, and more emphasis has been placed on the planning concept of “transformation” rather than “new construction/rebuilding,” with more emphasis on the inner development of cities, microrenewal, and adaptive improvement of urban space. Even in China, where the built-up area has grown exponentially over the past 30 years, spatial expansion is no longer the dominant form of urban development, and many cities are facing a shift from “incremental planning” to “stock planning” [16]. In China, in particular, a large number of small and medium-sized cities are now moving toward shrinking cities after urban expansion, with serious urban population loss and an increasing trend toward aging [17].

Concerned with the development of these cities, scholars and practitioners have proposed a range of urban regeneration and development strategies that can be summarized in three main development planning directions: regrowth, urban islands, and dedensification and greening [18]. Regardless of the development strategy, what needs to be acknowledged is the irreversibility of urban shrinkage, and that urban growth and decline, like life cycles, are seen as natural processes of urban change, requiring planning managers to shift a commonly accepted perception [19]. Scholars have suggested that local policymakers should examine whether sprawling urban landscapes meet the real needs of the current population and improve urban green space networks to increase the livability and attractiveness of cities, thereby mitigating population loss and enhancing economic vitality [20–22].

In contrast to China's Tier 1-2 cities, there is a large demand for healthcare in small and medium-sized cities that have undergone a brief and rapid urban expansion and are now gradually shrinking. Perhaps due to its unique natural environment and dietary habits, Northeast China has long been a region with a high prevalence of chronic diseases such as cardiovascular disease, diabetes, and gout [23, 24]. In many cities in Northeast China, there is widespread and strong awareness of the need for chronic disease patients to go outdoors for physical, mental, and spiritual healing [25]. Through ongoing observations and interviews over many years, this study considers that people with chronic illnesses in the region value rely more on outdoor blue, green, and white spaces for healing and therapy in appropriate seasonal and climatic conditions. In the cities of Jinzhou in Liaoning Province and Jiamusi in Heilongjiang Province, for example, outside of the extreme weather days in the northeast, large numbers of people regularly enter urban squares, parks, and waterfront streets where people can gather and relax on an almost daily basis to enjoy the healing and therapeutic effects of the landscape through a variety of health behaviors and activities. Therefore, as cities shift from sprawl to contraction and respond to the current needs of the population to continue to enhance the health and well-being of people with chronic diseases, the former suburban landscape needs to be reexamined not only for its therapeutic qualities but also for new place-making strategies in urban regeneration. However, much of the previous research has focused on examining the positive effects of landscape environments on patients [26, 27] and explaining the formation and evolution of therapeutic landscapes. Few scholars have integrated urban development and planning concepts in a public health context, examining the therapeutic nature of an evolving landscape and exploring how to shape therapeutic landscapes for sustainable well-being in urban regeneration.

**Figure 1:**
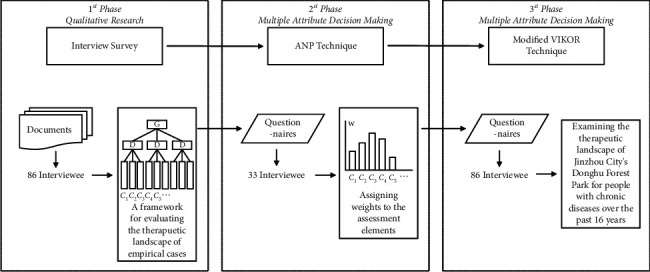
The process and design of this study.

In summary, this study uses the city of Jinzhou in Liaoning Province, China, as a case study. The city has experienced a brief and rapid urban expansion and is now shifting to a shrinking city with significant population loss and an aging trend. The city has a high prevalence of chronic diseases in the northeast and is generally representative of healthcare resources and the development of the built-up areas. The purpose of this study is to develop a framework for assessing the therapeutic nature of everyday urban landscapes based on the health care needs of people with chronic diseases, examine the therapeutic nature of the former Jinzhou Suburban Riverfront Forest Park as it has developed and evolved over the past 16 years, and explore strategies for place-making and regeneration of therapeutic landscapes for the well-being of people with chronic diseases from a dynamic impact and continuous development perspective. The design of this study is shown in Figure 1. Firstly, this study uses a literature review to initially extract the elements of therapeutic assessment for everyday urban landscapes and then, through focus group interviews, constructs a framework for assessing the therapeutic landscape of the East Lake Forest Park in Linghe District, Jinzhou City, based on the health care needs of people with chronic illnesses. Secondly, a network analysis method (ANP) was applied to assign weights to the assessment elements based on expert opinion, and a modified VIKOR technique was applied to examine the therapeutic landscape of Jinzhou City's Donghu Forest Park for people with chronic diseases over the past 16 years, in conjunction with interviews and questionnaires administered by the public. Finally, based on the results of the assessment and analysis, strategies for the creation and renewal of the therapeutic landscape of the East Lake Forest Park are explored in the context of Jinzhou's progressive move toward a shrinking city, in order to sustain the well-being of people with chronic diseases.

The remaining sections of this study are organized as follows. A review of the relevant literature is presented in Section 2. The research methodology and steps are clarified in Section 3. The findings and discussion are presented in Section 4, and the study is summarized in Section 5.

## 2. Literature Review: Healing and Therapeutic Landscape Design

The term “therapeutic landscape” is often used to describe this type of place space and has been defined by scholars as “a place that sustains physical and psychological recovery and comfort” [10]. Conradson [4] states that therapeutic landscapes involve a complex reciprocal relationship between a person and the broader social context of his or her environment. From the 1920s onwards, with the rapid development of modern medical technology in the form of drugs and medical diagnostic equipment, which revolutionized the way humans were treated, the potential of the landscape environment to regulate and restore disease has rarely been mentioned or studied. It was not until the late 1980s that scholars argued that medicine should be based on harmony between people and their environment, and numerous studies have since confirmed that natural landscapes can promote human health in both physical and psychological terms, with the therapeutic effects being more spiritual in nature. Duff [28] explicitly recommends that those suffering from mental illness go to places with proven therapeutic properties for ongoing healing. A “therapeutic” landscape is not just one type of space; that is, a landscape environment that can have a therapeutic effect includes many different types of spaces, such as waterfront spaces [7, 29], villages [4], horticulture and gardens [30], natural forests [5], and courtyards [31]. Due to the more complex components of the landscape environment and the service functions it can provide, its therapeutic function is not only spiritual. Moreover, the healing of landscape environments is not only for people with illnesses but also for people from all walks of life and all age groups [26]. Finlay et al. [14] divide the therapeutic landscape into two components: blue space and green space, and discuss the different effects they have on older people in their old age. Blue space is particularly important for mental recovery and building mental health, while green space can have a positive impact on older people's social activities. As such, research on the therapeutic nature of such spaces is often focused on spatial environments such as natural landscapes, landscape gardens, residential courtyards, and built environments [32–34].

Gesler [35] describes the sense of place in therapeutic landscapes and the four main dimensions: the natural environment, the built environment, the symbolic environment, and the social environment. In the context of related theoretical perspectives (e.g., Aesthetic Affective Theory (AAT) and spiritual evolutionary theory), scholars have summarized three main characteristics of therapeutic landscapes and therapeutic gardens: relief of physical symptoms, illness or trauma, reduction of stress for individuals dealing with emotionally and/or physically stressful experiences, and improvement of overall well-being [36, 37]. Therapeutic landscapes within healthcare buildings include places where horticultural therapeutic activities exist such as therapeutic gardens, healing gardens, meditation gardens, and memorial gardens [38]. However, this relational perspective on therapeutic landscapes makes it difficult to know how to shape places to enhance well-being, and how place experiences may be healing or therapeutic has been undertheorized [39–41]. The research carried out by Doughty [42] and Pitt [41] highlights that exploring the role of the moving body in interacting with the natural environment provides valuable insight into how places act therapeutically, and they both argue for a more dynamic understanding of Butterfield and Martin [43], which also discussed how the sensory richness of place provides opportunities for therapeutic effects to emerge when studying the environment of cancer support centers. Gorman [44] provided more insight into exactly how these sensory experiences become therapeutic by focusing on one particular sense. He explored the role of smell within Community Supported Agricultural projects. Belčáková et al. [13] proposed a strategy for greening hospital facilities and external spaces and adding elements that highlight and support the therapeutic effects of the spatial environment, creating specific therapeutic landscape design forms that create the required physical environmental base for patients.

## 3. Research Methodology and Steps

This study uses a multicriteria decision-making approach (MCDM) to structure the research design. Through a review of the relevant literature, an initial assessment framework for assessing the therapeutic nature of everyday urban landscapes was extracted. A focus group approach was then used to collect qualitative data and to identify the assessment framework (dimensions/elements) in this study through qualitative inductive analysis based on the well-being and health of people with chronic diseases and to clarify the interactions between the factors in the structure of the assessment framework. The ANP technique was then used to assign weights to the assessment factors through the administration of a questionnaire. In combination with the people's interviews and questionnaires, the modified VIKOR technique was applied to examine the therapeutic landscape of Jinzhou East Lake Forest Park for people with chronic illnesses over the last 16 years of its development and evolution. In summary, the results of each phase of the study were integrated to explore the evolution of the therapeutic landscape in Jinzhou East Lake Forest Park and to explore strategies for the creation and renewal of the therapeutic landscape in East Lake Forest Park in order to continue to promote the well-being of patients with chronic illnesses.

### 3.1. Focus Group Interview Method and Data Collection

Focus group interviewing originated in 1941 at Columbia University's Division of Radio Studies, when Paul Lazarsfeld invited Robert Merton to help assess listeners' reactions to radio programs. The method is designed to gather information of interest to the researcher and tends to uncover a wide range of opinions from a variety of types of people. It provides a more natural context than individual interviews, where the researcher is the presenter, the listener, the observer, and finally the analyst who processes the data using an inductive approach. It is in the focus group that the researcher gains understanding based on the content of the discussion, rather than aiming to test preconceived theories or hypotheses. Importantly, the focus group approach allows the researcher to observe the interaction of the participants and to capture the substance of the views, opinions, experience, and attitudes expressed in the spoken word, as well as the design of the discussion questions to assess the issues related to the research topic.

This study considers the behavioral and perceptual impact of respondents' own chronic disease categories, as well as the slow walking distance from the forest park due to the development and expansion of the urban built environment, on their behavior and perception in the therapeutic landscape. Before sampling, the chronic diseases that are most prevalent in the area were classified into three categories: cardiovascular, metabolic, and respiratory diseases [45]. The three types of sampling areas were divided into a radius of 5-, 15-, and 30-minute walking distance from each park entrance. Respondents were recruited anonymously in these three types of areas, and those who entered the focus group interviews were required to be chronically ill, have outdoor health habits, and enter East Lake Forest Park at least three days a week on average to receive healing and therapeutic influences from the daily landscape. Respondents were recruited and placed into focus groups according to their subdistrict and chronic disease category. A total of 9 groups of 8 to 12 people each were interviewed, making a total of 86 people. After all the qualitative data had been collated and analyzed, it was found that sufficient information had been obtained on the research themes, so the number of focus groups was not increased (information on the respondents is shown in Table 1).

### 3.2. ANP Technique and Data Collection

Given that the elements used to examine the everyday therapeutic nature of urban landscapes have different attribute categories, the analytical approach is based on the Analytic Network Process (ANP) used in multicriteria decision analysis [46]. Compared to older generations of multicriteria decision analysis (MCDA) methods (e.g., AHP), the ANP method is more accurate because it has the ability to examine relationships between variables. In addition to its multicriteria analysis capabilities, the ANP technique does not require independence among the criteria and factors; thus, it can be used as an efficient method for cases wherein the criteria are interdependent and their factors affect the final decision-making goal. The procedure for the ANP technique consists of a series of following steps:

Step (1): model (network) construction.
Step (2): design questionnaire and survey.
Step (3): (1) construct pairwise comparison weight; (2) calculate criteria weight, using (1) and (2); (3) do testing of consistency; the value of consensus can be estimated by (3).
  (1) $$\begin{array}{c} A={\left[a_{ij}\right]}_{n\times n}=\begin{array}{c} \vdots \\ C_{i}\\ \vdots \\ C_{n} \end{array}\overset{C_{1}\begin{array}{cc} C_{j} & ... \end{array}}{\left[\begin{array}{ccccc} \cdots & \frac{w_{1}}{w_{j}} & \cdots & \frac{w_{1}}{w_{n}}\\ \vdots & \vdots & \vdots \\ \frac{w_{i}}{w_{1}} & \cdots & \frac{w_{i}}{w_{j}} & \cdots & \frac{w_{i}}{w_{n}}\\ \vdots & \vdots & \vdots \\ \frac{w_{n}}{w_{1}} & \cdots & \frac{w_{n}}{w_{j}} & \cdots & \frac{w_{n}}{w_{n}} \end{array}\right]}, \end{array}$$
  (2) $$\begin{array}{c} r_{i}={\left(\prod_{j=1}^{n}a_{ij}\right)}^{1/n}, \end{array}$$
  (3) $$\begin{array}{c} C.I.=\frac{\left(\lambda_{\max}-n\right)}{\left(n-1\right)},\\ C.R.=\frac{C.I.}{R.I.}. \end{array}$$
Step A4: construct an unweighted supermatrix.
Step A5: weighted supermatrix.
Step A6: limited supermatrix.

In this study, the ANP questionnaire was administered to people with chronic illnesses who had previously participated in focus group interviews and who had lived in Jinzhou for over16 years and entered East Lake Forest Park almost daily. A total of 33 respondents completed the questionnaire, and 28 valid questionnaires were returned.

### 3.3. Modified VIKOR Technique and Data Collection

VIKOR (VlseKriterijumska Optimizacija I Kompromisno Resenje) is a multicriterion decision-making method proposed by Professor Serafim Opricovic and Professor Gwo-Hshiung Tzeng in 1998 whereas VIKOR is one of the optimal compromises of multicriteria decision-making methods, whose basic idea is to first define the positive-ideal solution (optimal solution) and the negative-ideal solution (the worst solution).

Step V1: determine the best values and the worst values (very bad← 0, 1, 2,…, 10⟶ very good).
Step V2: compute the gap values of group utility and the gap values of individual regret.
Step V3: summarize the gap between group utility and individual regret. These values can be calculated using
  (4) $$\begin{array}{c} s_{k}=\sum_{j=1}^{n}w_{j}r_{kj}=\sum_{j=1}^{n}w_{j}\frac{\left(\left|f_{j}^{\text{aspired}}-f_{kj}\right|\right)}{\left(\left|f_{j}^{\text{aspired}}-f_{j}^{\text{worst}}\right|\right)}. \end{array}$$
Step V4: rank the alternatives.

A therapeutic landscape performance assessment questionnaire was administered to respondents who participated in focus group interviews in Jinzhou City's Donghu Forest Park. Respondents were asked to evaluate the therapeutic landscape performance of the park based on their knowledge and perceptions of the park and the development of the park over the past 16 years, as collected by this study. Finally, a total of 86 questionnaires were distributed, and 81 valid questionnaires were returned.

## 4. Results and Discussion

### 4.1. Constructing Therapeutic Landscape Assessment Frameworks and Influence Network Relationships

This study explores the role of sensory and embodied place experiences as an area of significant contribution, using the elements of the everyday urban landscape that can contribute to the well-being and therapeutic care of people with chronic illness as the basic questions for semistructured interviews in focus groups. Sensory and embodied experiences of place contribute to an understanding of how a place may act therapeutically [34, 41, 43]. Group discussions in the focus group interviews have been centered on the questions “how does East Lake Forest Park heal and treat you” and “what elements of the landscape are healing in.” The interviews were transcribed into verbatim transcripts, and qualitative data was summarized and analyzed in Nvivo12 software. This study used the general qualitative inductive analysis method proposed by David R Thomas in 2006 to analyze the data. The analysis procedure of this method can be described as coding of qualitative information into a number of “concept nodes,” which are “categorized” and formed organizationally. The expected outcome of this process is the creation of three to eight summary categories that capture the key aspects of the themes identified in the raw data. The inductive coding that identifies more than eight main categories is incomplete, in which case certain categories are combined or the assessor identifies which themes or categories are most important.

**Figure 2:**
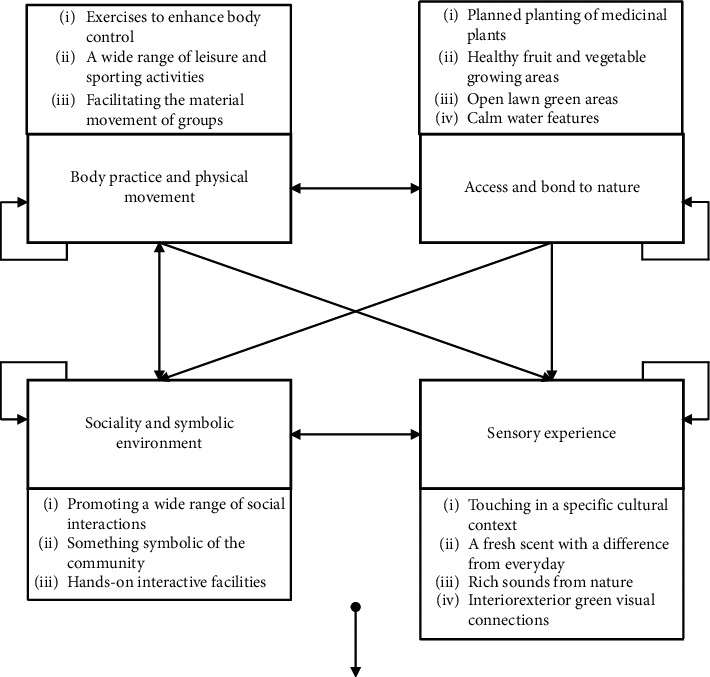
Assessment framework and influence network relationships.

A memo has been written for each category, such as association, connection, and meaning. Each category was revised and improved through continuous comparative analysis by searching for new concepts and contradictions. After the results were organizationally formed to be sufficiently stable and saturated, the results of the inductive analysis are shown in Figure 2. Four main categories were identified, and the relationships of their interaction networks were clarified. Each category has a self-influencing relationship due to the interactions between the concept nodes under the category. Within this network of influence, there are three sets of two-way influence relationships within the main categories named “body practice and physical movement” and “access and bond to nature,” “body practice and physical movement” and “sociality and symbolic environment,” and “sociality and symbolic environment” and “sensory experience.” Unidirectional influences include the following: both “body practice and physical movement” and “access and bond to nature” have an impact on “sensory experience.” “Sociality and symbolic environment” receive the influence from “access and bond to nature.” As shown in Figure 2, the 14 conceptual nodes fall under four main categories and can be considered as a hierarchical structure. In this study, the main categories obtained through the inductive analysis are considered as assessment dimensions, and the coded conceptual nodes are considered as assessment elements, thus building an assessment framework for the therapeutic landscape of Jinzhou East Lake Forest Park.

### 4.2. Weighting Analysis of Assessment Elements in the Healing Landscape

In this study, the ANP technique is applied to calculate the relative weights of each assessment element based on the examination of the influence network relationships between the assessment constructs. After passing the consistency check, the unweighted supermatrix constructed in this study is shown in Table 2. After integrating the empirical judgments of 28 respondents and the limited supermatrix, the weights of each assessment dimension/element are shown in Table 3. The results show that sociality and symbolic environment (*D*_3_) and sensory experience (*D*_4_) are the two constructs with the highest relative importance. The remaining levels of importance are, in order of importance, *D*_1_ and *D*_2_. Under the *D*_3_ dimension, the assessment element with the highest local weight is promoting a wide range of social interactions (*C*_8_). In this assessment framework, the relative weights of the assessment elements are closer to each other under *D*_1_, the lowest relative weight.

This means that the outdoor social opportunities, symbolic environment, and range of sensory experiences that East Lake Forest Park can provide are of paramount importance in enhancing the well-being and health of local people with chronic illnesses. As suggested by scholars in previous studies, healing and therapeutic landscapes should be integrated with active living in the course of urban development, linking them to social interactions and together creating a supportive environment that is essential for healthy living [47, 48]. In addition, positive and influential social culture, people, or a spiritual intention related to the therapeutic nature of the landscape can also have an important healing and therapeutic effect on groups of people with chronic illnesses. In certain contexts, people with chronic illnesses may even emulate the behavior of the symbolic environment and actively restore their self-confidence [8]. Some of the sensory experiences that the East Lake Forest Park offers to the public that are different from everyday urban life are also important landscape elements for people with chronic illnesses, for example, fresh scents [44], culturally appropriate touch [49], and nature-derived sound and visual stimuli [50].

### 4.3. Landscape Evolutionary Development and Therapeutic Assessment in Jinzhou East Lake Forest Park

With the rapid urbanization of the whole of China, the built environment of Jinzhou City has been upgraded, and new residential developments have been built on both sides of the Xiaoling River, the main river flowing through the built-up area of the city. Over the past 20 years, a number of high-quality residential projects have been launched in the area, and local policymakers have set the long-term goal of creating a “livable and healthy” residential area. In this context, the slow walking trail system in the East Lake Forest Park and on both sides of the Xiaoling River has developed over the years into a major urban park landscape, enhancing the well-being of Jinzhou residents and providing daily outdoor fitness and social interaction for a wide range of people. According to the site survey, the East Lake Forest Park has gone through three key stages of development, from the initial construction in 2004 to the gradual shaping of the landscape in 2012 and then to the completion and refinement in 2020 (as shown in Table 4). Therefore, this study examines the development and evolution of the therapeutic landscape of the East Lake Forest Park in Jinzhou City by using these three development periods as time points and assesses the therapeutic nature of this everyday urban landscape with the goal of continuously enhancing the well-being and health care of people with chronic diseases.

In 2004, Jinzhou East Lake Park was only constructed with walking paths, the trees in the park were not yet cultivated or were still in the growing stage, and the infrastructure was lacking. In 2012, the third year prior to the completion of the construction work on the East Lake Forest Park, the vegetation coverage was much higher than when construction began in 2004, the roads were better, and the infrastructure was evenly distributed throughout the park. From the west side of the park to the middle of the park, three areas have been built 200 meters apart for the public to relax and enjoy. In the middle of the park, starting from the riverfront on the southern side of the site, three more viewing spots have been built along the riverbank to the east. By 2020, five years after the completion of East Lake Park, the car park and cultural square on the west side of the park had been built, the road leading from the entrance to the river had been widened, the size of each recreational space in the park had been increased, the central rest area had been transformed into a comprehensive sports ground with football, basketball, and badminton courts, and the park had introduced the water of the Xiaoling River through the entire park, further enhancing its ornamental qualities. The park has been further enhanced by the introduction of the Xiaoling River. The east side of the park is less covered with vegetation than the west side, and the area between the roads is mostly grass. The distribution of trees in the park is more rational than in 2012, with different types of trees planted depending on their location, giving the park a more structured appearance overall.

The results of the analysis of the therapeutic landscape assessment in different development and construction periods show that the healing and therapeutic effects of Jinzhou East Lake Forest Park for the local chronic disease patient group are continuing to improve year by year as the quality of the built environment improves. However, at the level of the assessed components, the performance of the *D*_1_ and *D*_3_ components improved significantly; however, the *D*_2_ and *D*_4_ components showed varying degrees of decline in points. This implies that the access and exposure to the natural environment dimension in the East Lake Forest Park have not improved with the built environment from the perspective of the chronically ill, and even the open green space is decreasing, and medicinal plants are not being used sufficiently in the vegetation community planning. Therefore, this study suggests that the current East Lake Forest Park needs to stimulate and improve the senses of people with chronic illnesses who enter the site through certain colors, rhythmic sounds, directional smells, operable interactive facilities, etc., so that they can detach themselves from their negative or undesirable state. In addition, this study suggests an attempt to make medicinal plants the basic plants of the entire planting community. Using a large number of plants to create visual amenities, public services can be placed near the medicinal planting community. In addition, referring to the influence networks in Figure 1, this study argues that the social and symbolic nature of the urban landscape can be used to achieve some kind of spiritual healing through activities, services, and even rituals, creating a so-called therapeutic encounter, which is a potential function not revealed in past concepts of community design, a process of psychological well-being and health through “participation.” This also responds to the complex process of healing taking place in the aforementioned contexts mentioned by W. M. Gesler, which are not just spatially generated therapeutic outcomes but are more often dependent on the relational interactions that arise in these contexts, some kind of empowerment, inclusion or, as mentioned above, collective social behavior, and the following of symbolic imagery.

On the other hand, this study recommends that local policymakers continue to promote the regeneration and quality of the East Lake Forest Park by designing different types of privacy and social spaces and turning the unused green space into an accessible rehabilitation garden that can be used by the elderly and chronically ill to provide appropriate physiotherapy and even light exercise, for example, by providing gravel paths to balance the rehabilitation therapy walking exercises. Appropriately placed fruit and vegetable growing areas within the grounds also allow for the production of safe, nutritious vegetables. This study proposes a reconstruction of the building with greenhouses. Its function should be updated. In the past, the greenhouse had everything people need for herbs. It could also be utilized during wintertime. After renovation, chronic disease patients can cultivate healthy fruits and vegetables suitable for nutrition here. Fig trees or citrus plants can also be cultivated along with different kinds of herbs having a variety of smells and tastes (for example, basil, horsemint, bee balm, basil thyme, chive, or meadow sage). While growing herbs, patients can improve their management and responsibility skills as well as their interest in the natural environment. These activities can also enable them to gain better social skills and communication in a team. Additionally, such activity makes patients less stressed during their rehabilitation therapy, and they can feel themselves be more efficient and successful. What needs to be maintained and further enhanced is the development of the Jinzhou East Lake Forest Park as an area that offers good group mobility to a wide range of people. The concept of continuous “flow” is important in a therapeutic landscape where one may be so fully engaged in physical activity that different concerns and stresses may be temporarily forgotten. As Doughty [42] found, group walking can be seen as a supportive social space through shared movement and social relationships within the environment.

## 5. Conclusions

This study provides useful insights for examining the therapeutic nature of everyday urban landscapes as they evolve over the course of urban development and for planning landscape development to enhance people's well-being. Firstly, based on a review of the relevant literature, a systematic assessment framework was developed that can be used to assess the therapeutic nature of everyday urban landscapes, and then, a hierarchical assessment framework was constructed based on the qualitative data collected through focus group interviews and inductive analysis. The ANP technique was then applied to train the relative weights of the components/elements in the assessment framework. Finally, the modified VIKOR technique was applied to determine the value of the gap between the actual level and the desired level of Jinzhou East Lake Forest Park in 2004, 2012, and 2020 as the assessment cases. This study found that the healing and therapeutic effect of Jinzhou's East Lake Forest Park on the local chronic disease patient population has been increasing year by year. The park has been developed over the years and is suitable for a variety of recreational sports, group exercise, and mobility and a wide range of social interaction activities. In addition to this, the healing and positive therapeutic benefits that the case can unleash on people with chronic illnesses and its landscape nodes already have some degree of symbolic impact. However, compared to 16 years ago, the level of access and contact with the natural environment in the East Lake Forest Park has not improved with the built environment, and even the open green spaces are declining, and medicinal plants are not being used enough in the vegetation community planning. Therefore, this study suggests that future development planning for Jinzhou East Lake Forest Park should focus on a more microscopic landscape design level, designing different types of open green spaces, improving the utilization of unused green spaces, and providing the public with a richer sensory experience through certain colors, rhythmic sounds, and directional smells. For local policymakers, this study provides an effective way to examine the therapeutic nature of everyday urban landscapes as urban sprawl shifts to contraction and to explore planning for the continued development of therapeutic landscapes in relation to networks of influence between landscape elements.

The limitations of this study should be acknowledged, as they may provide guidance for future research. Respondents recruited for this study were from a broad group of people with chronic diseases earning socially acceptable wages, and the analysis does not apply to people with chronic diseases who are of higher socioeconomic status or who live in different geographical locations and climates. In addition, elements of the therapeutic landscape of the East Lake Forest Park in 2004 and 2012 can only be discussed and generalized based on recollections of recruited respondents and personal experience judgments in focus group interviews. The quantitative techniques applied in this study were additive in nature during the performance evaluation phase of the empirical cases, which may have detracted from reality in the ranking and selection of performance. In future research, nonadditive quantitative techniques (e.g., fuzzy integral method) could be attempted to conduct performance assessment analysis of multiperiod therapeutic landscapes. It is important to acknowledge that the therapeutic landscape development planning strategies explored in this study are specific to Jinzhou City Forest Park and are not applicable to everyday urban landscapes in other areas. However, the assessment methodology provided in this study and the assessment framework constructed within a decision management perspective may provide a theoretical basis and a key reference for future examination of the therapeutic nature of urban landscapes in other regions.

## Figures and Tables

**Table 1:** Basic information about participants in the focus group interviews.

Respondent source	Chronic disease category			Age (year)		
	Cardiovascular	Metabolic	Respiratory	20–35	35–50	50–65
A. Residents within a 5-minute slow walking distance	8	10	10	7	15	6
B. Residents within a 15-minute slow walking distance	8	12	8	5	14	9
C. Residents within a 30-minute slow walking distance	12	10	8	4	11	15
Total (people)	28	32	26	16	40	30

**Table 2:** Construct unweighted supermatrix.

	*C*_1_	*C*_2_	*C*_3_	*C*_4_	*C*_5_	*C*_6_	*C*_7_	*C*_8_	*C*_9_	*C*_10_	*C*_11_	*C*_12_	*C*_13_	C_14_
*C*_1_	1.00	0.00	0.00	0.20	0.30	0.18	0.35	0.20	0.17	0.47	0.00	0.00	0.00	0.00
*C*_2_	0.00	1.00	0.00	0.15	0.53	0.33	0.40	0.24	0.44	0.15	0.00	0.00	0.00	0.00
*C*_3_	0.00	0.00	1.00	0.65	0.17	0.49	0.25	0.56	0.39	0.38	0.00	0.00	0.00	0.00
*C*_4_	0.13	0.15	0.05	1.00	0.00	0.00	0.00	0.00	0.00	0.00	0.00	0.00	0.00	0.00
*C*_5_	0.25	0.10	0.17	0.00	1.00	0.00	0.00	0.00	0.00	0.00	0.00	0.00	0.00	0.00
*C*_6_	0.36	0.48	0.55	0.00	0.00	1.00	0.00	0.00	0.00	0.00	0.00	0.00	0.00	0.00
*C*_7_	0.26	0.27	0.23	0.00	0.00	0.00	1.00	0.00	0.00	0.00	0.00	0.00	0.00	0.00
*C*_8_	0.28	0.45	0.69	0.25	0.45	0.50	0.47	1.00	0.00	0.00	0.57	0.25	0.36	0.45
*C*_9_	0.15	0.33	0.25	0.63	0.18	0.16	0.38	0.00	1.00	0.00	0.21	0.55	0.54	0.28
*C*_10_	0.57	0.22	0.06	0.12	0.37	0.34	0.15	0.00	0.00	1.00	0.22	0.20	0.10	0.27
*C*_11_	0.42	0.25	0.08	0.22	0.30	0.22	0.23	0.09	0.25	0.55	1.00	0.00	0.00	0.00
*C*_12_	0.08	0.10	0.35	0.45	0.42	0.25	0.24	0.25	0.40	0.03	0.00	1.00	0.00	0.00
*C*_13_	0.15	0.27	0.25	0.06	0.12	0.15	0.40	0.41	0.30	0.15	0.00	0.00	1.00	0.00
*C*_14_	0.35	0.38	0.32	0.27	0.16	0.38	0.13	0.25	0.05	0.27	0.00	0.00	0.00	1.00
SUM	4.00	4.00	4.00	4.00	4.00	4.00	4.00	3.00	3.00	3.00	2.00	2.00	2.00	2.00

**Table 3:** Weighting of the assessed dimensions/elements.

Dimensions	Local weights	Elements	Local weights	Global weights
Body practice and physical movement (*D*_1_)	0.188	Exercises to enhance body control (*C*_1_)	0.250	0.046
		A wide range of leisure and sporting activities (*C*_2_)	0.300	0.056
		Facilitating the material movement of groups (*C*_3_)	0.450	0.085

Access and bond to nature (*D*_2_)	0.062	Planned planting of medicinal plants (*C*_4_)	0.100	0.006
		Healthy fruit and vegetable growing areas (*C*_5_)	0.170	0.011
		Open lawn green areas (*C*_6_)	0.480	0.030
		Calm water features (*C*_7_)	0.250	0.016

Sociality and symbolic environment (*D*_3_)	0.375	Promoting a wide range of social interactions (*C*_8_)	0.430	0.162
		Something symbolic of the community (*C*_9_)	0.360	0.135
		Hands-on interactive facilities (*C*_10_)	0.210	0.078

Sensory experience (*D*_4_)	0.375	Touching in a specific cultural context (*C*_11_)	0.240	0.090
		A fresh scent with a difference from every day (*C*_12_)	0.250	0.091
		Rich sounds from nature (*C*_13_)	0.280	0.106
		Interior exterior green visual connections (*C*_14_)	0.230	0.088

**Table 4:** Assessment of therapeutic landscapes based on well-being and health care for people with chronic diseases.

2004	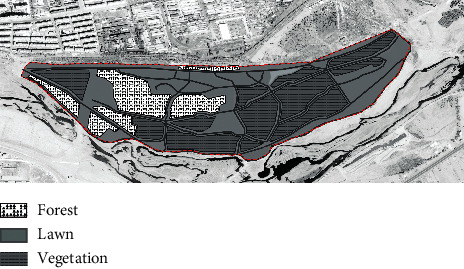													

Dimensions	Body practice and physical movement (*D*_1_)			Access and bond to nature (*D*_2_)				Sociality and symbolic environment (*D*_3_)			Sensory experience (*D*_4_)			

Gap ratio	0.68			0.57				0.86			0.44			

Elements	*C*_1_	*C*_2_	*C*_3_	*C*_4_	*C*_5_	*C*_6_	*C*_7_	*C*_8_	*C*_9_	*C*_10_	*C*_11_	*C*_12_	*C*_13_	*C*_14_

Gap ratio	0.93	0.43	0.71	0.97	0.74	0.38	0.68	0.78	0.93	0.92	0.96	0.24	0.20	0.40

Total performance: 3.44														

2012	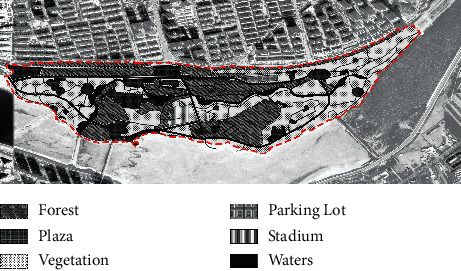													

Dimensions	Body practice and physical movement (*D*_1_)			Access and bond to nature (*D*_2_)				Sociality and symbolic environment (*D*_3_)			Sensory experience (*D*_4_)			

Gap ratio	0.54			0.60				0.55			0.58			

Elements	*C*_1_	*C*_2_	*C*_3_	*C*_4_	*C*_5_	*C*_6_	*C*_7_	*C*_8_	*C*_9_	*C*_10_	*C*_11_	*C*_12_	*C*_13_	*C*_14_

Gap ratio	0.60	0.68	0.42	0.68	0.57	0.69	0.43	0.44	0.64	0.59	0.88	0.56	0.50	0.41

Total performance: 4.35														

2020	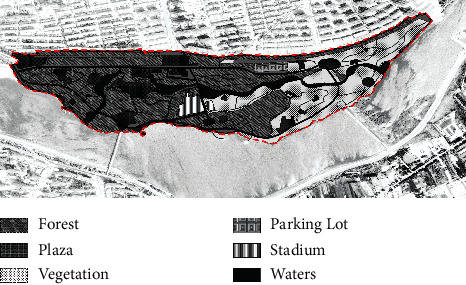													

Dimensions	Body practice and physical movement (*D*_1_)			Access and bond to nature (*D*_2_)				Sociality and symbolic environment (*D*_3_)			Sensory experience (*D*_4_)			

Gap ratio	0.36			0.56				0.42			0.46			

Elements	*C*_1_	*C*_2_	*C*_3_	*C*_4_	*C*_5_	*C*_6_	*C*_7_	*C*_8_	*C*_9_	*C*_10_	*C*_11_	*C*_12_	*C*_13_	*C*_14_

Gap ratio	0.43	0.36	0.31	0.53	0.37	0.72	0.39	0.31	0.56	0.42	0.80	0.42	0.38	0.28

Total performance: 5.64														

## Data Availability

The data used to support the findings of this study are included within the article.
